# The Acetylcholine-Activated Potassium Current Inhibitor XAF-1407 Terminates Persistent Atrial Fibrillation in Goats

**DOI:** 10.3389/fphar.2020.608410

**Published:** 2021-01-27

**Authors:** Vladimír Sobota, Giulia Gatta, Arne van Hunnik, Iris van Tuijn, Marion Kuiper, James Milnes, Thomas Jespersen, Ulrich Schotten, Sander Verheule

**Affiliations:** ^1^ Department of Physiology, Cardiovascular Research Institute Maastricht, Maastricht, Netherlands; ^2^ Xention Ltd., Cambridge, United Kingdom; ^3^ Department of Biomedical Sciences, Faculty of Health and Medical Sciences, University of Copenhagen, Copenhagen, Denmark

**Keywords:** atrial fibrillation, acetylcholine-activated potassium channel, cardioversion, mapping, remodeling

## Abstract

**Aims:** The acetylcholine-activated inward rectifier potassium current (I_KACh_) has been proposed as an atrial-selective target for the treatment of atrial fibrillation (AF). Using a novel selective I_KACh_ inhibitor XAF-1407, the study investigates the effect of I_KACh_ inhibition in goats with pacing-induced, short-term AF.

**Methods:** Ten goats (57 ± 5 kg) were instrumented with pericardial electrodes. Electrophysiological parameters were assessed at baseline and during intravenous infusion of XAF-1407 (0.3, 3.0 mg/kg) in conscious animals before and after 2 days of electrically induced AF. Following a further 2 weeks of sustained AF, cardioversion was attempted with either XAF-1407 (0.3 followed by 3 mg/kg) or with vernakalant (3.7 followed by 4.5 mg/kg), an antiarrhythmic drug that inhibits the fast sodium current and several potassium currents. During a final open chest experiment, 249 unipolar electrograms were recorded on each atrium to construct activation patterns and AF cardioversion was attempted with XAF-1407.

**Results:** XAF-1407 prolonged atrial effective refractory period by 36 ms (45%) and 71 ms (87%) (0.3 and 3.0 mg/kg, respectively; pacing cycle length 400 ms, 2 days of AF-induced remodeling) and showed higher cardioversion efficacy than vernakalant (8/9 vs. 5/9). XAF-1407 caused a minor decrease in the number of waves per AF cycle in the last seconds prior to cardioversion. Administration of XAF-1407 was associated with a modest increase in QTc (<10%). No ventricular proarrhythmic events were observed.

**Conclusion:** XAF-1407 showed an antiarrhythmic effect in a goat model of AF. The study indicates that I_KACh_ represents an interesting therapeutic target for treatment of AF. To assess the efficacy of XAF-1407 in later time points of AF-induced remodeling, follow-up studies with longer period of AF maintenance would be necessary.

## Introduction

Atrial fibrillation (AF) is the most common clinically diagnosed cardiac arrhythmia, affecting approximately 3% of the population aged 20 years and older (Kirchhof et al., 2016). It is associated with increased risk for stroke and death (Kirchhof et al., 2016). AF causes both electrical and structural remodeling of the atria (Schotten et al., 2011). The rapid process of electrical remodeling (hours to days) entails shortening of the atrial effective refractory period (aERP), whereas the much slower process of structural remodeling (weeks to years) refers to alterations in atrial tissue structure. Both processes contribute to the gradual progression of AF, to increasing AF episode duration and to decreasing the efficacy of antiarrhythmic drugs. Furthermore, antiarrhythmic drug treatment is hampered by significant adverse effects, including ventricular proarrhythmia. Thus, there is an unmet need for development of new antiarrhythmic therapies that are atrial-specific, with fewer side effects.

The acetylcholine-activated potassium current (I_KACh_) is an inward rectifier that contributes to stabilization of the resting membrane potential and phase three repolarization (Milnes et al., 2012). I_KACh_ is predominantly present in atria (Koumi and Wasserstrom, 1994), sinoatrial node (Noma and Trautwein, 1978) and atrioventricular (AV) node (Sakmann et al., 1983). Constitutively active I_KACh,_ i.e. I_KACh_ that is active in the absence of acetylcholine, has been reported in patients with chronic AF, suggesting an increased contribution to the total inward rectifier K^+^ current in atria with AF-induced remodeling (Dobrev et al., 2005). Selective I_KACh_ inhibition by tertiapin reversed the shortening of action potential duration after AF-induced remodeling in patients with chronic AF (Dobrev et al., 2005), making I_KACh_ an interesting atrial-selective target for treatment of AF.

Recently, a new antiarrhythmic compound XAF-1407 (3-methyl-1-[5-phenyl-4-[4-(2-pyrrolidin-1-ylethoxymethyl)-1-piperidyl] thieno [2,3-days]pyrimidin-6-yl]azetidin-3-ol) was developed by Xention (Xention Ltd., United Kingdom), showing high selectivity for I_KACh_ (Fenner et al., 2020). Compared to other major ion channels in the heart, the selectivity of XAF-1407 to I_KACh_ channels was shown to be at least 1,000 fold higher (Fenner et al., 2020). In this study we aimed to investigate the cardioversion efficacy of XAF-1407 and its effects on atrial refractoriness and other electrophysiological parameters. We have used a goat model of pacing-induced AF that recapitulates the AF-induced atrial remodeling also observed in patients (Wijffels et al., 1995).

## Materials and Methods

### Animal Model

The research protocol was approved by the local ethical board for animal experimentation and is in compliance with the European directive 2010/63/EU on the protection of animals used for scientific purposes.

Ten female Dutch milk goats from a local breeder with body weights of 57 ± 5 (range 47–64) kg and age 24 ± 3 (range 20–27) months were used in the study. The animals were housed individually in conventional cages of approximately 8 m^2^ with straw bedding and constant temperature and humidity. The animals were kept at a 12 h light/dark cycle with a free access to food (hay and pellets) and water. Animal welfare was checked daily and body weight measured once a week. The goat model of pacing-induced AF has been chosen because it is well characterized and has proven to provide valuable insights into the mechanisms underlying AF (Wijffels et al., 1995; van Hunnik et al., 2018). It has been previously used to study the antiarrhythmic effect of drugs for treatment of AF (Blaauw et al., 2004; van Hunnik et al., 2016; Ji et al., 2017; van Hunnik et al., 2018). Female goats have been used in order to allow comparability with previous studies that were solely based on female goats (Blaauw et al., 2004; Blaauw et al., 2007; Verheule et al., 2010; van Hunnik et al., 2016; Ji et al., 2017; van Hunnik et al., 2018). Other reasons for the use of female goats was their tranquil nature and a very limited availability of male goats.

A left-sided thoracotomy was performed under general anesthesia that was initiated with sodium thiopental (20 mg/kg) and maintained with isoflurane (0.5%), sufentanyl (6 μg/kg/h i.v.) and propofol (5–10 μg/kg/h i.v.). A custom-build patch of electrodes was sutured epicardially under the left atrial (LA) appendage through small incision in pericardium. A plaque of sensing electrodes was implanted pericardially above the LA, together with the electrodes of the implanted stimulator (Medtronic Itrel®, Medtronic, Minneapolis, MN). Furthermore, a pair of sensing electrodes was sutured on the pericardium above both the left and the right ventricular free wall. Three silver discs (2 cm diameter) were implanted subcutaneously and were used as reference electrodes. The wires of all custom-build electrodes were incorporated in a single cable, tunneled subcutaneously to the neck and exteriorized. The animals were treated post-operatively with antibiotics and received analgesia with buprenofine (5–10 µg/kg i.m. twice a day, first day after the surgery) and carprofen (2-4 mg/kg s.c., first three days after surgery). A recovery period of 2–3 weeks was allowed before the first experiment.

### Effects of XAF-1407 on Cardiac Electrophysiology in Conscious Animals

**FIGURE 1:**
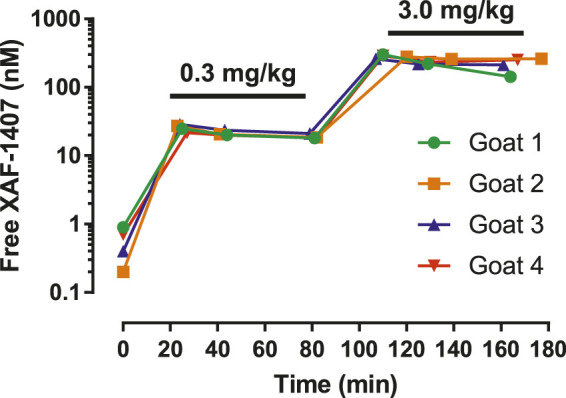
Free plasma concentration of XAF-1407 during the experiments in conscious goats. The data from four animals show that the dosing regimen reached stable plasma concentration of XAF-1407 in both applied doses. Time 0 corresponds with the beginning of the intravenous infusion.

The effect of XAF-1407 was tested during pacing protocols in five conscious animals in control conditions (normal atria) and after 2 days of AF (electrically remodeled atria). The drug was administered intravenously, using sodium acetate buffer (0.1 mol/l) as a vehicle. Two doses of XAF-1407 were investigated: 0.3 and 3.0 mg/kg, infused as a bolus over 20 min (15 and 135 μg/kg/min for the doses of 0.3 and 3.0 mg/kg, respectively). To maintain stable plasma level throughout the study protocol, a 1-h maintenance infusion (5.9 and 59 μg/kg/min for the doses of 0.3 and 3.0 mg/kg, respectively) was started after the bolus. Multiple plasma samples were taken during the drug administration, allowing assessment of XAF-1407 concentration in free plasma by liquid chromatography-mass spectrometry. The dosing regimen was designed to reach stable plasma levels of XAF-1407 during electrophysiological measurements, as shown in Figure 1. The dose of 0.3 mg/kg corresponds to the lower range of maximum effectivity while the dose of 3.0 mg/kg represents a safe and effective dose of XAF-1407 in the horse (Fenner et al., 2020).

Basic electrophysiological parameters were determined in normal atria and after 2 days of AF (electrically remodeled atria). AF was induced and maintained by the implanted stimulator, applying 50 Hz burst stimulation every other second. The aERP was determined using an S1-S2 protocol. The Wenckebach cycle length (the cycle length at which a second degree AV block occurs) was determined using an S1-S1 protocol, applying cycle lengths from 500 to 200 ms with decremental steps of 50 ms. Both atrial and ventricular unipolar electrograms were recorded during the measurements together with a pseudo-ECG obtained from the implanted electrodes. A period of at least 7 days followed the measurements in electrically remodeled atria to allow complete reverse remodeling of the atria. To investigate the effect of vagal activity on the I_KACh_ inhibition by XAF-1407, atropine (0.04 mg/kg) was administered intravenously in two animals with non-remodeled atria, followed by a dose of 3.0 mg/kg of XAF-1407.

### Cardioversion Attempts in Conscious Animals

A period of at least 7 days followed the measurements in electrically remodeled atria to allow complete reverse remodeling of the atria (Schotten et al., 2003). Subsequently, AF was induced again and maintained for 3 weeks. Cardioversion attempts with XAF-1407 (dose of 0.3 mg/kg followed by 3.0 mg/kg were performed after 2 weeks of AF maintenance. For comparison, we used vernakalant, an antiarrhythmic drug for treatment of AF that we have recently tested in the goat model of pacing-induced AF (van Hunnik et al., 2016; van Hunnik et al., 2018). Vernakalant inhibits fast sodium current and several potassium currents, including the transient outward current (I_to_) and has been recently approved for European market (Fedida et al., 2005; Camm, 2014). The dosing regimen of vernakalant (dose of 3.7 mg/kg followed by 4.5 mg/kg) was designed to reach plasma concentrations of 3 μg/ml (the dose of 3.7 mg/kg) and 5 μg/ml (the dose of 4.5 mg/kg), which are the plasma levels that are known to be effective in patients (van Hunnik et al., 2018). Each dose was administered using a loading dose infused over 20 min, followed by a maintenance dose for another 20 min. After drug administration, all animals were monitored during a wash-out period of 30 min. The order of the cardioversion attempts was randomized, yielding the same average AF duration for each drug. One animal did not develop persistent AF and was excluded from the analysis of cardioversion efficacy.

### Open Chest Experiment

A final open chest experiment was performed 23–24 days after the AF initiation. Anesthesia was induced with sodium thiopental (20 mg/kg) and maintained with sufentanyl (6 μg/kg/min i.v.), propofol (5-10 mg/kg/h i.v.) and rocuronium (0.3 mg/kg/h i.v.). The heart was exposed through a left-sided thoracotomy. Mapping electrode arrays (249 electrodes, interelectrode distance 2.4 mm, array diameter 4 cm) were placed on both the LA and the right atrial (RA) free wall to allow recording of unipolar atrial electrograms (sampling rate 1 kHz, 16-bit resolution, filter bandwidth 0.1–400 Hz). Standard limb leads were used to record the surface ECG. After instrumentation, a stabilization period of at least 30 min was allowed before the start of the measurements. Left ventricular pressures were measured using a pressure-volume catheter (Sentron Europe BV, Roden, The Netherlands). Two ten-pole catheters, positioned in the coronary sinus and along the right atrial free wall were connected to an external defibrillator (Physio-Control Lifepak 9B, Medtronic, Minneapolis, Minnesota, United States) to allow electrical cardioversion of AF.

Before the drug infusion, the heart was endocardially cardioverted by a DC shock (≤20 J) to assess aERP (S1-S2 protocol; pacing output 4x threshold current) and conduction velocity (S1-S1 protocol; 500–200 ms, pacing output 2x threshold current). AF was re-induced by burst pacing and XAF-1407 was administered, applying an identical dosing regimen as the one that was used during electrophysiological measurements in conscious animals. Unipolar electrograms were recorded during the drug administration to allow later assessment of changes in AF properties. Conduction velocity (CV) and aERP were reassessed for each dose of XAF-1407. Once the experiment was completed, the animal was euthanized by rapid excision of the heart.

### Data and Statistical Analysis

The atrial fibrillation cycle length (AFCL) was calculated using a median window of 60 s. Pseudo-ECG signals were analyzed manually using the IDEEQ software (Instrument Development Engineering and Evaluation, Maastricht University, Maastricht, The Netherlands). Corrected QT intervals (QTc) were calculated by using a goat-specific version of Bazzet’s formula, QTc = QT/(RR)^0.6^ (Mohan et al., 2009).

Custom-made software in the MATLAB programming environment (The MathWorks, Inc., Natick, Massachusetts, United States) was used for analysis of unipolar atrial electrograms. Local atrial activations were determined using a probabilistic annotation algorithm (Zeemering et al., 2012). The following measures of AF substrate complexity were calculated: median AF cycle length, maximum wave size, number of waves per AF cycle, number of breakthrough waves per AF cycle, fractionation index and maximum dissociation (Zeemering et al., 2012). For both atria, the AF properties were assessed at baseline (intervals of 60 s), prior to cardioversion (intervals of 1.5–2 s) and in a preceding interval of 10 s. Atrial wavelength was calculated as a product of aERP and CV (Wiener and Rosenblueth, 1946).

Data were statistically analyzed using IBM SPSS Statistics for Macintosh, version 25 (IBM Corp., Armonk, N.Y., United States) and GraphPad Prism version 7.03 (GraphPad Software, La Jolla, CA, United States). A log-rank test was used to compare the cardioversion efficacy of XAF-1407 with vernakalant and the change in Wenckebach cycle length. A Friedman’s test was used to compare changes in the left-ventricular hemodynamic parameters and in ECG parameters. AFCL prolongation was compared using Mann-Whitney test. A linear mixed-effects model was used for the comparison of drug-induced changes in conduction velocity. Two-way repeated measures ANOVA was used for the comparison of the changes in aERP and wavelength. The data are presented as mean ± standard deviation unless stated otherwise. The percentual changes in ECG parameters are stated as median and interquartile range. A *p*-value < 0.05 was considered as statistically significant.

## Results

**FIGURE 2:**
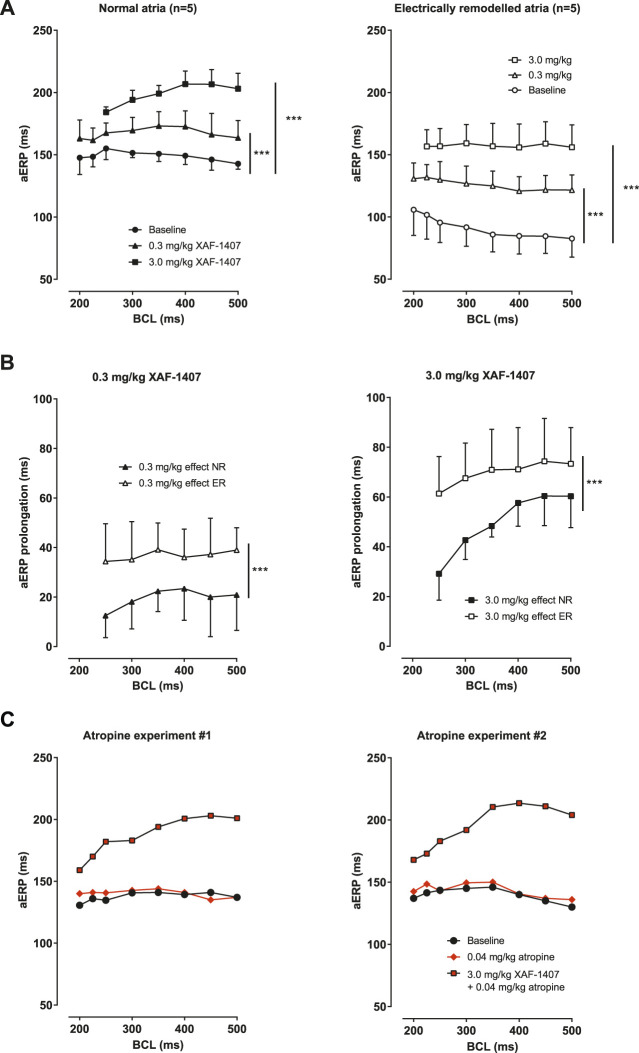
The effect of XAF-1407 on atrial refractoriness. **(A)** Prolongation of aERP at all burst cycle lengths (BCL) was observed in normal (*N* = 5) and electrically remodeled atria (*N* = 5) after the administration of 0.3 and 3.0 mg/kg of XAF-1407. **(B)** Compared to normal atria, the effect of XAF-1407 was larger after electrical remodeling for both applied doses of the drug. **(C)** Additional experiments with atropine showed that vagal inhibition does not alter the effect of XAF-1407 on atrial refractoriness. Mixed model, ***p < 0.001.

A dose-dependent prolongation of aERP in LA was observed after the administration of XAF-1407 in normal atria as well as in electrically remodeled atria at all pacing rates (Figure 2A). The aERP prolongation caused by XAF-1407 was generally smaller at shorter cycle lengths (reverse rate dependence). The effect of XAF-1407 was larger in electrically remodeled atria than in normal atria (Figure 2B). To investigate how I_KACh_ inhibition is affected by vagal tone, we performed aERP measurements after vagal inhibition by atropine in two normal goats without electrical remodeling. The measurements showed that atropine does not alter the efficacy of XAF-1407 to prolong aERP, as shown in Figure 2C.

**FIGURE 3:**
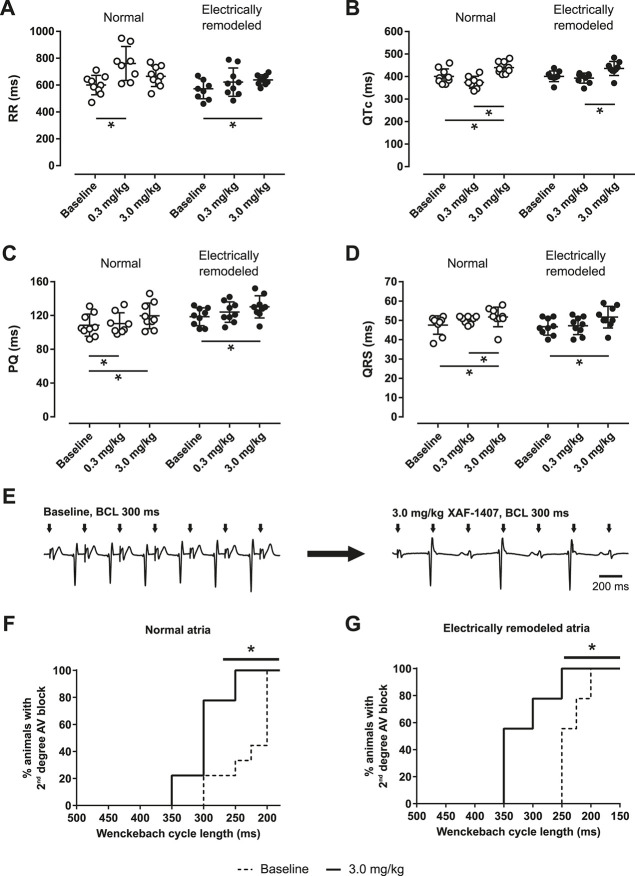
Changes in electrophysiological parameters during administration of XAF-1407 in conscious animals. Prolongation of RR **(A)**, QTc **(B)**, PQ **(C)** and QRS **(D)** intervals was observed in goats with normal atria and electrically remodeled atria (*N* = 9). Statistical comparisons were made between baseline and the doses of 0.3 and 3.0 mg/kg of XAF-1407. Friedman’s test, *p < 0.05. An example of electrocardiogram demonstrating LA pacing (BCL 300 ms) during baseline and after the administration of 3.0 mg/kg of XAF-1407 illustrate the presence of second degree AV block after the drug administration **(E)**. Prolongation of Wenckebach cycle length was observed after the administration of 3.0 mg/kg of XAF-1407 in normal atria **(F)** as well as in electrically remodeled atria **(G)**. Log-rank test, *p < 0.05, *N* = 9.

A prolongation of RR interval was observed after the administration of XAF-1407 (Figure 3A). In the animals without electrical remodeling, a prolongation of RR interval by 25% (18–30%) was observed after the administration of 0.3 mg/kg of XAF-1407 (Figure 3A). Contrary to that, the effect of the same dose after 2 days of pacing-induced AF was only limited, with a prolongation of 4% (3–11%). The dose of 3.0 mg/kg of XAF-1407 resulted in a prolongation of RR interval by 12% (2–13%) and 14% (3–18%) in the hearts with and without pacing-induced AF, respectively. Similarly, a small prolongation of PQ by 10% (7–11%) in normal atria and by 4% (3–18%) in electrically remodeled atria was observed after the administration of 3.0 mg/kg of XAF-1407 (Figure 3C). The effect of XAF-1407 on ventricular electrophysiology was limited, with the QRS prolongation in normal atria by 7% (6–11%) and in electrically remodeled atria by 8% (7–11%) after 3.0 mg/kg of XAF-1407 (Figure 3D). The same dose of XAF-1407 caused the prolongation of QTc by 8% (5–13%) and 9% (6–11%) in normal and electrically remodeled atria, respectively. (Figure 3B). No ventricular proarrhythmic events (extrasystoles, non-sustained ventricular tachycardia) were observed during administration of either vernakalant or XAF-1407. To further explore the effect of I_KACh_ inhibition on the AV node, the Wenckebach cycle length was investigated in awake animals during atrial pacing. The median Wenckebach cycle length was 200 ms in the animals without any history of AF and 250 ms after atrial electrical remodeling. At 3.0 mg/kg of XAF-1407 the Wenckebach cycle length prolonged to 300 and 350 ms, respectively (Figures 3E–G).

**FIGURE 4:**
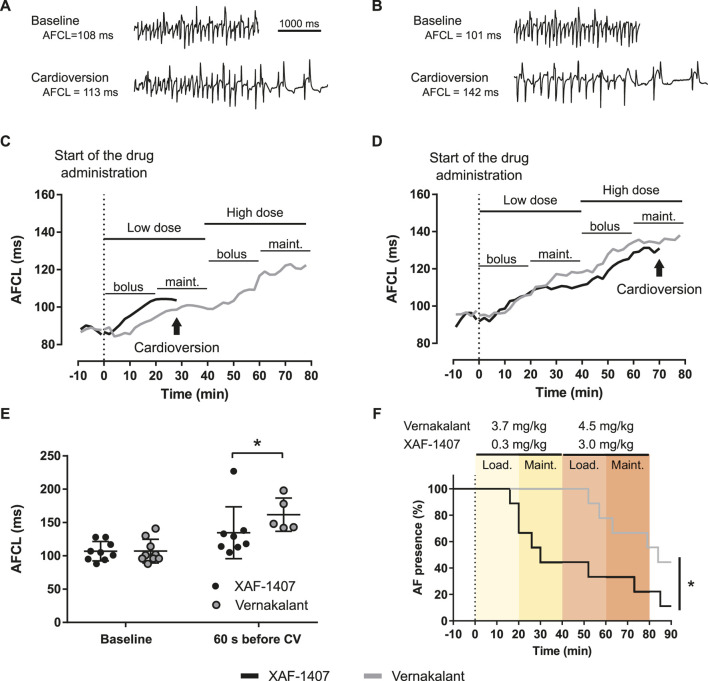
Atrial fibrillation cycle length (AFCL) prolongation and cardioversion during administration of XAF-1407 and vernakalant. **(A)** Left atrial electrograms illustrating the fast and irregular rhythm during AF at baseline and 60 s before cardioversion with XAF-1407 and **(B)** vernakalant. **(C)** Examples of gradual prolongation of AFCL during the administration of XAF-1407 with cardioversion occurring during the maintenance phase after the dose of 0.3 mg/kg and **(D)** 3.0 mg/kg of XAF-1407. Prolongation of AFCL during the cardioversion attempt with vernakalant in the same animal is depicted for comparison. **(E)** Comparison of AFCL at baseline (*N* = 9) and 60 s before cardioversion (*N* = 8 and *N* = 5 for XAF-1407 and vernakalant, respectively). Mann Whitney test, *p < 0.05. **(F)** XAF-1407 showed higher cardioversion efficacy than vernakalant (*p < 0.05, Log-rank test, *N* = 9). The loading and maintenance phases of the applied dosing are highlighted.

Pharmacological cardioversion of AF with either XAF-1407 or vernakalant was attempted in awake animals after 16 ± 2 days of AF maintenance. One animal did not develop stable AF and was excluded from the analysis of cardioversion efficacy. Infusion of XAF-1407 resulted in cardioversion in eight out of nine goats (5 cardioversions with the dose of 0.3 mg/kg, 2 with the dose of 3.0 mg/kg, one during washout) with an average time to cardioversion of 40 ± 26 min (Figure 4F). Compared to that, five out of nine goats cardioverted during the infusion of vernakalant (zero with the dose of 3.7 mg/kg, four cardioversions with the dose of 4.5 mg/kg, one during washout) with an average time to cardioversion of 67 ± 14 min. Administration of both XAF-1407 and vernakalant resulted in gradual prolongation of AFCL (Figures 4A–D). Compared to the administration of vernakalant, the AFCL just prior to cardioversion was shorter for XAF-1407 than for vernakalant (Figure 4E).

**FIGURE 5:**
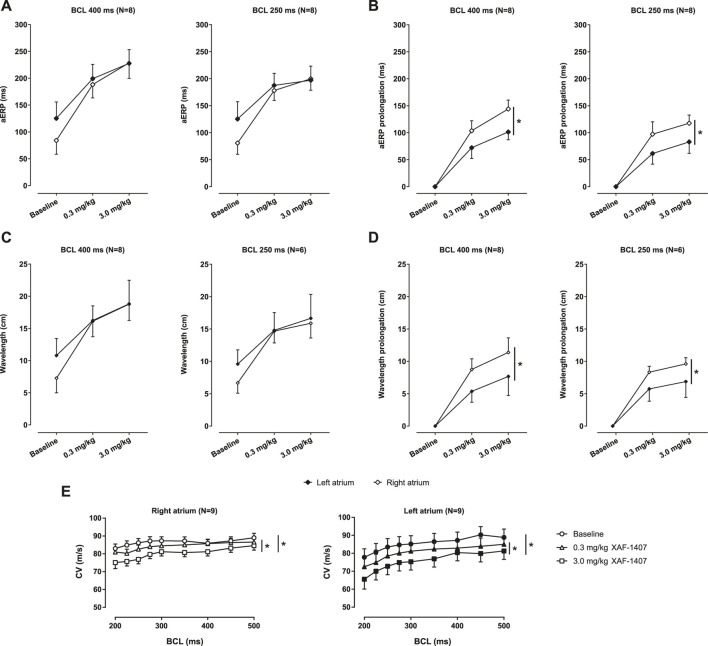
The effect of XAF-1407 on atrial refractoriness, wavelength and conduction velocity during pacing. **(A)** XAF-1407 induced a dose-dependent prolongation of atrial effective refractory period (aERP). **(B)** The degree of aERP prolongation was larger in the right atrium. **(C)** A strong, dose-dependent prolongation of wavelength was observed in both atria during pacing. **(D)** Similarly, the prolongation of wavelength was more pronounced in right atrium. For aERP and wavelength, the sample sizes are stated in the brackets next to the burst cycle length (BCL). **(E)** Dose-dependent decrease of conduction velocity was observed in both atria. Presented as estimated mean ± standard error of the mean. *p < 0.05.

The open chest experiment was performed in nine animals. One animal developed sterile pericarditis and was excluded from the experiment. The measurements of aERPs revealed that the effect of XAF-1407 on atrial refractoriness was generally larger in the RA compared to the LA. The aERPs at baseline were typically shorter in the RA when compared to the LA (Figure 5A). The dose of 0.3 mg/kg of XAF-1407 caused a strong prolongation in aERP in both RA and LA (Figure 5B). The dose of 3.0 mg/kg resulted in further prolongation of aERP in both atria. Similarly, the administration of XAF-1407 led to a strong, dose-dependent prolongation of wavelength in both atria (Figure 5C) with a more pronounced effect in the right atrium (Figure 5D). The administration of XAF-1407 led to a dose-dependent decrease of CV at all cycle lengths in LA and RA with no apparent rate-dependent differences (Figure 5E).

Administration of XAF-1407 had only a modest effect on ventricular hemodynamics (Table 1). The left ventricular (LV) end-diastolic and systolic pressure showed a minor increase. Similarly, there was a small increase in the maximum rate of LV pressure change (dP/dt_max_) after the administration of XAF-1407.

**TABLE 1:** Hemodynamic parameters in left ventricle.

	Baseline	XAF-1407	
		0.3 mg/kg	3.0 mg/kg
*P*_enddia_, mmHg	5.19 (3.85–10.65)	6.69 (5.71–10.27)^a^	7.84 (5.33–10.66)^a^
*P*_sys_, mmHg	116 (93-125)	120 (109–130)	123 (114–126)
*dP*/*dt*_max_, mmHg/s	3,672 (3,105–4,334)	4,183 (3,689–4,426)	4,286 (3,599–5,118)

Data are presented as median and interquartile range. *P*_enddia_ end-diastolic pressure, *P*_max_ maximum pressure, *dP*/*dt*_max_ peak rate of pressure.

^a^ *p* < 0.05, compared to baseline, *N* = 8.

**FIGURE 6:**
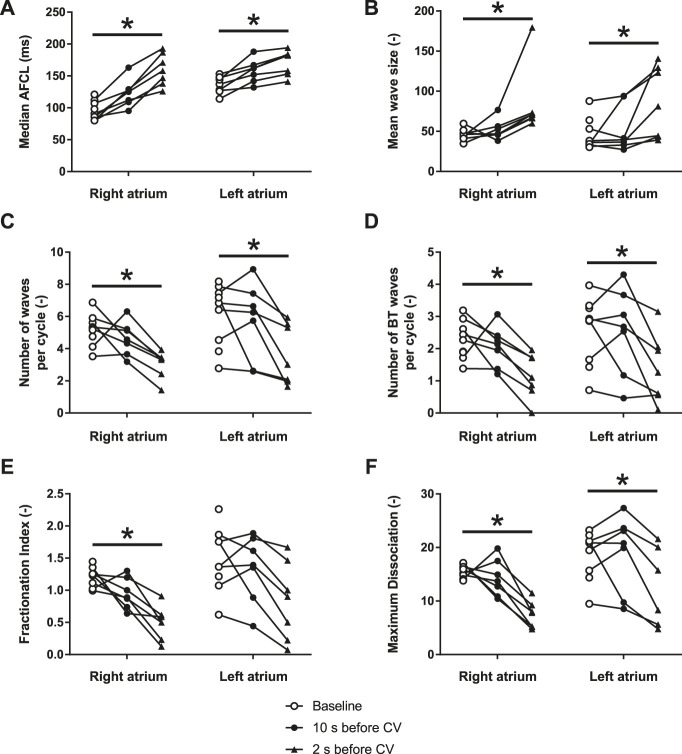
XAF-1407 reduces the complexity of atrial fibrillation in LA and RA. **(A)** Median atrial fibrillation cycle length (AFCL), **(B)** mean wave size, expressed as a number of electrodes assigned to one fibrillatory wave, **(C)** number of waves per AF cycle, **(D)** number of breakthrough waves per AF cycle, **(E)** fractionation index and, **(F)** maximum wave dissociation. Friedman’s test, *p < 0.05, *N* = 9.

**FIGURE 7:**
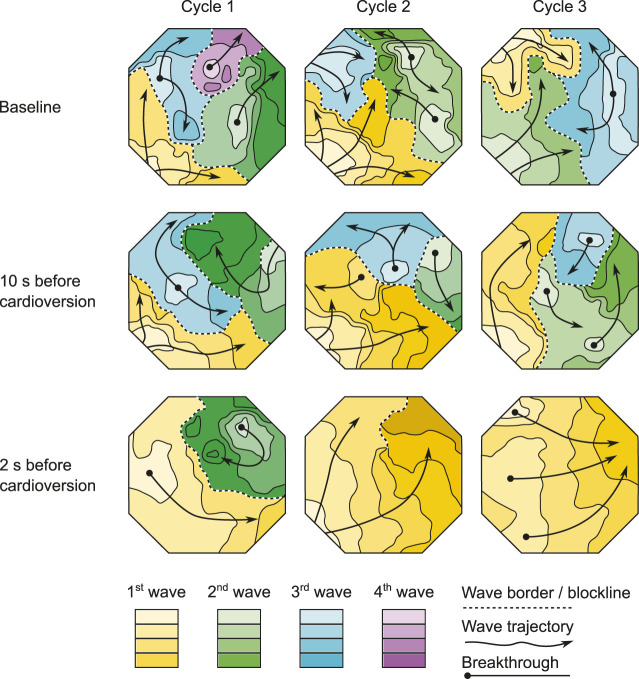
The effect of XAF-1407 on fibrillation patterns. Wave maps of three consecutive beats at baseline, 10 s before cardioversion and 2 s before cardioversion are shown. Each map displays the territory of individual fibrillation waves within the mapped area of LA free wall during a single AF cycle. The color-coding depicts the sequence of wave appearance. The isochronal lines within waves are separated by 10 ms. AF complexity decreased 1.5–2 s before pharmacological cardioversion with XAF-1407. However, there was only a moderate reduction in AF complexity in the last 10 s preceding this rapid organization period.

During the 1.5–2 s before pharmacological cardioversion with XAF-1407, AF complexity decreased in both atria (Figures 6, 7). This period of organization was associated with decrease in activation frequency, less electrical dyssynchrony and fewer breakthrough waves. Yet, there was only a minor reduction of AF complexity in the last 10 s preceding this rapid organization period, indicating that the decline in AF complexity only occurred just before cardioversion.

## Discussion

This study demonstrates that selective I_KACh_ inhibition by XAF-1407 represents a safe therapeutic strategy for treatment of AF. We showed that selective inhibition of I_KACh_ by XAF-1407 results in prolongation of aERP and AFCL leading to AF termination. XAF-1407 caused a modest prolongation of QRS and QTc, in the absence of any observed ventricular proarrhythmic effects.

### Effect of XAF-1407 on Atrial Refractoriness

The aERP prolongation after the administration of XAF-1407 was substantially larger in electrically remodeled atria. This finding supports the hypothesis that constitutively active I_KACh_ increases during AF (Dobrev et al., 2005). Reverse rate-dependency in the effect of XAF-1407 was observed in normal atria as well as in electrically remodeled atria. Similar findings have been reported in the goat model of AF for AVE0118, a class III antiarrhythmic drug inhibiting the ultra-rapid delayed rectifier (I_Kur_) and transient outward (I_to_) currents (Blaauw et al., 2004). Prolongation of aERP has also been reported for XAF-1407 in an equine model of AF (Fenner et al., 2020).

The effect of XAF-1407 on atrial refractoriness in normal atria was relatively large and unexpected. To investigate the possibility that a high vagal tone in normal goats augments the aERP prolongation after I_KACh_ inhibition, we performed an additional experiment. In two goats without AF-induced electrical remodeling, we measured aERPs after vagal inhibition by atropine, followed by I_KACh_ inhibition by XAF-1407. Although the number of animals in this experimental group was small, the absence of any aERP shortening after the infusion of atropine strongly suggests that the observed effect of XAF-1407 on refractoriness in normal atria was not caused by high vagal tone. We therefore propose that the effect of XAF-1407 on atrial refractoriness in normal goat atria can be explained by constitutively active I_KACh_ even without AF-induced electrical remodeling. Such finding of constitutively active I_KACh_ have been reported for normal canine atria (Voigt et al., 2008) but is clearly not the case in human atria in the absence of AF-induced remodeling (Dobrev et al., 2005).

The effect of I_KACh_ inhibition on atrial refractoriness was larger in the RA than in the LA, with baseline aERPs in the RA being generally shorter after 4 weeks of pacing-induced AF. The administration of 0.3 mg/kg of XAF-1407 decreased the right-to-left aERP gradient and the gradient entirely disappeared after the dose of 3.0 mg/kg. It has been shown that the gradient in I_KACh_ density between the atria can differ among species (Anumonwo and Lopatin, 2010). Therefore, we suggest that the larger effect of XAF-1407 in the RA might be caused by a stronger contribution of I_KACh_ to repolarization in goat RA. Compared to XAF-1407, the effect of vernakalant on atrial refractoriness was less pronounced, as described by previous studies in the goat model of pacing-induced AF (van Hunnik et al., 2016; van Hunnik et al., 2018). Selective I_KACh_ inhibition therefore appears to have a strong effect on atrial refractoriness in goats.

### Effect of XAF-1407 on Atrial Conduction and Wavelength

We observed a moderate dose-dependent reduction of conduction velocity during the infusion of XAF-1407, indicating a class I effect of the drug, possibly mediated through a depolarization of the resting membrane potential, resulting in the decreased number of available sodium channels. The effect was comparable between RA and LA. In comparison, even more pronounced, dose-dependent decrease in atrial conduction velocity was observed for vernakalant, which is known to exhibit a class I effect due to the inhibition of the fast sodium current (Fedida et al., 2005; van Hunnik et al., 2016).

The observed strong effect of XAF-1407 on atrial refractoriness and its moderate effect on atrial conduction velocity resulted in substantial prolongation of atrial wavelength in both atria which is a finding that confirms the proposed class III action of the compound. In contrast, in a previous study we observed that vernakalant does not lead to a prolongation of atrial wavelength in electrically remodeled atria (van Hunnik et al., 2016). Although the direct comparison of the degree of wavelength prolongation is not possible because the measurements with XAF-1407 were performed under general anesthesia while the measurements with vernakalant were performed in awake animals, our findings indicate strong effect of XAF-1407 on prolongation of atrial wavelength, suggesting strong efficacy of the drug to terminate AF.

### Cardioversion Efficacy of XAF-1407

The efficacy of XAF-1407 to cardiovert AF (8 out of 9, 89%) was comparable to the efficacy reported in an equine model of persistent AF (80% and 70% after 11 and 17 days of persistent AF; respectively) (Fenner et al., 2020). It was also similar to the efficacy of previously tested class III antiarrhythmic drugs in the same goat model of pacing-induced AF. The administration of I_Kur_ and I_to_ inhibitor AVE0118 resulted in cardioversion rate of 63% when AF was induced for 53 ± 19 days (Blaauw et al., 2004) and 100% when AVE0118 was administered in combination with a rapid delayed rectifier current (I_Kr_) inhibitors dofetilide or ibutilide in goats with AF induced for 57 ± 7 days (Blaauw et al., 2007). For the inward rectifier potassium current (I_K1_) inhibitor PA-6 the cardioversion rate after 3 weeks of AF was 83% (Ji et al., 2017). Although direct comparison of cardioversion efficacies within the same goat model is not straightforward due to the different time points investigated and the dosing regimens used, our results indicate that I_KACh_ inhibition is a promising therapeutic strategy for treatment of AF.

The effect of the vehicle (0.1 mol/l sodium acetate buffer) on cardioversion efficacy has not been investigated in this study. However, previous experimental study with XAF-1407 reported a 0% cardioversion rate after intravenous administration of the vehicle only (Fenner et al., 2020).

Considering the similar time point of AF development, we argue that the efficacy of XAF-1407 to cardiovert AF is superior to vernakalant. The efficacy of vernakalant to cardiovert AF (5 out of 9, 56%) was identical to the previous study in goats with 11 days of AF in which the same cardioversion rate (56%) was achieved (van Hunnik et al., 2016).

### XAF-1407 Slows Down the Conduction of Sinoatrial and Atrioventricular Node

It has been reported that I_KACh_ channels are expressed in sinoatrial (SA) (Noma and Trautwein, 1978) and atrioventricular (AV) node (Sakmann et al., 1983). In the present study, we show that I_KACh_ inhibition by XAF-1407 had mild bradycardic effect during sinus rhythm in normal atria at a dose of 0.3 mg/kg. A stronger deceleration of sinus rhythm in electrically remodeled atria was observed after the dose of 3.0 mg/kg. To study the effect of I_KACh_ inhibition on the AV node, we investigated the shift in Wenckebach cycle length, showing that infusion of XAF-1407 markedly increased the median Wenckebach cycle length. This observation is also in agreement with the observed prolongation of PQ interval after the infusion of 3.0 mg/kg of XAF-1407. Contrary to our findings, enhanced AV node conduction and small increase in heart rate were observed when XAF-1407 was administered in horses (Fenner et al., 2020). The different effect between goats and horses might be possibly explained by a species-specific expression of ion channels in the SA and AV node. The design of our study did not allow us to investigate the mechanisms underlying the changes in the nodal function due to the administration of XAF-1407 in goats. The observed effect on SA and AV node does not have to be caused by the direct effect of the compound on the nodal cells, but it might be also the result of the coupling of nodal cells with atrial cardiomyocytes or due to changes in electrotonic interactions (Jalife, 1984).

Our data show that I_KACh_ inhibition by XAF-1407 affects the function of the SA and AV nodes. In the context of AF, deceleration of sinus rhythm can potentially be seen as an adverse effect. Slowing down of sinus rhythm increases the probability of dispersion of atrial refractoriness and occurrence of atrial ectopy, both of which potentially facilitate reinitiation of AF. By contrast, decreasing of AV nodal conduction during AF may potentially be beneficial because it contributes to the reduction of ventricular rate during AF, thereby possibly preventing tachycardia-induced cardiomyopathy.

### XAF-1407 Decreases AF Complexity

The progressive character of AF-induced remodeling makes the fibrillation pattern more complex and the arrhythmia more persistent (Verheule et al., 2010). The current study showed a decrease of AF complexity 1.5–2 s prior to cardioversion during XAF-1407 administration. This time period was associated with a decrease in the total number of waves and breakthrough waves per AF cycle, as well as with an increase of wave size. A similar decrease of AF complexity has been described in the same animal model for the I_K1_ inhibitor PA-6 (Ji et al., 2017). Only limited changes in AF pattern- and complexity-related parameters were observed in the period of 2–12 s prior to cardioversion. This finding indicates that the decrease of AF complexity 1.5–2 s prior to cardioversion is associated with the cardioversion process itself rather than caused by the I_KACh_ inhibition by XAF-1407. Similar finding has been reported for a class I antiarrhythmic drug cibenzoline in which an abrupt increase of AFCL and a reduced occurrence of fractionated electrograms in the last beats preceding cardioversion has been observed (Shan et al., 2004).

### Ventricular Safety of XAF-1407

A modest effect of XAF-1407 on LV pressure was observed in anesthetized animals. The administration of XAF-1407 resulted in a small increase in end-diastolic pressure and a small increase of LV systolic pressure and its maximum derivative (dP/dt_max_). Due to the fact that the hemodynamic measurements were performed during an open-chest procedure, their interpretation should be performed with caution.

Administration of 0.3 mg/kg of XAF-1407 was not associated with prolongation of QTc and QRS and the prolongation of both QTc and QRS after the infusion of 3.0 mg/kg of XAF-1407 was only limited. The small effect of XAF-1407 on QTc prolongation together with an absence of ventricular proarrhythmic events (extrasystoles, non-sustained ventricular tachycardia) during its administration indicate ventricular safety of the drug. Considering the high selectivity of XAF-1407 for I_KACh_ channels (Fenner et al., 2020) and the fact that the median prolongation of QTc and QRS did not exceed 10%, the results suggest that XAF-1407 is a selective inhibitor of I_KACh_ in the goat model of AF.

### I_KACh_ as a Target for AF Treatment in Humans

There are several studies that investigated IKACh inhibition as a target for treatment of AF in humans (Walfridsson et al., 2015; Podd et al., 2016). Modestly selective IKACh inhibitors AZD2927 and A7071 increased atrial refractoriness and were associated with AF termination in dogs (Walfridsson et al., 2015). However, these positive results could not been replicated in atrial flutter patients (Walfridsson et al., 2015). Similarly, the highly-selective IKACh blocker BMS914392 did not show any reduction in AF burden in patients with paroxysmal AF, although it could terminate AF in a dog model (Podd et al., 2016).

In contrast to BMS914392, AZD2927 and A7071, XAF-1407 has been shown to inhibit both K_ir_3.1/3.4 heterotetramers, and K_ir_3.4/3.4 homotetramers with similar potency which has been shown to increase the antiarrhythmic efficacy of the compound (Fenner et al., 2020). Nevertheless, there are no data available yet on XAF-1407 in humans to verify the antiarrhythmic efficacy of XAF-1407 in patients with AF.

### Limitations

The current study represents an early time point in the development of AF-induced remodeling which is known to gradually increase AF stability over a time course of months (Verheule et al., 2010). A loss of efficacy during AF progression has been observed for several antiarrhythmic drugs in the goat model of AF (Verheule et al., 2010). To assess the efficacy of XAF-1407 in later time points of AF-induced remodeling, follow-up studies with longer period of AF maintenance will be necessary.

It has been shown that I_KACh_ contributes to the increased inward-rectifying potassium current in patients with chronic AF (Dobrev et al., 2005). Although our results indicate that I_KACh_ is also constitutively active in normal goat atria, our data does not preclude the effectiveness of I_KACh_ blockade as an antiarrhythmic treatment. Indeed, if I_KACh_ inhibition selectively prolongs atrial action potential duration in the absence of AF-induced electrical remodeling, its antiarrhythmic properties could be effective in a wider range of clinical settings and patient populations.

The work by Fenner et al. demonstrated that XAF-1407 is a highly selective inhibitor of I_KACh_ channels (Fenner et al., 2020). The goat model of pacing-induced AF that was used in this study did not allow us to investigate selectivity of the drug to different ionic currents. Therefore, it was not possible for us to describe the mechanisms underlying some of the observed effects of XAF-1407 on cardiac electrophysiology. The large effect of XAF-1407 on aERP in non-remodeled atria, its class I effect indicated by the decreased atrial conduction as well as the changes in the nodal function and the effect on ventricular electrophysiology described by the prolongation of QRS and QTc can be possibly explained by a non-specific effect of XAF-1407. Additional patch clamp experiments would be necessary to explain these effects of XAF-1407 on cardiac electrophysiology.

## Conclusion

Our study demonstrates that I_KACh_ inhibitor XAF-1407 has high efficacy to terminate AF, with pronounced atrial-selectivity. Our study in the goat model of AF thus indicates that I_KACh_ represents a promising therapeutic target for treatment of AF.

## Data Availability Statement

The raw data supporting the conclusions of this article will be made available by the authors, without undue reservation.

## Ethics Statement

The animal study was reviewed and approved by Animal Ethics Committee of Maastricht University.

## Author Contribution

VS, AvH, US, and SV designed the study. VS, GG, MK, and AvH planned and performed the experiments. VS, GG, IvT, and AvH analyzed the data. VS, GG, AvH, JM, TJ, US, and SV interpreted the data. VS and SV wrote the manuscript. All authors revised the manuscript and approved its final version.

## Funding

This project received funding from the European Union’s Horizon 2020 research and innovation programme under the Marie Sklodowska-Curie grant agreement No. 675351 and from the Netherlands Heart Foundation (CVON 2014-09, RACE V Reappraisal of Atrial Fibrillation: Interaction between hyperCoagulability, Electrical remodeling, and Vascular Destabilization in the Progression of Atrial Fibrillation).

## Conflict of Interest

GG is an employee of Medtronic BV. JM is a former employee of Xention Ltd. US received research grants from Roche and EP Solutions. He also received speaker fees from Johnson & Johnson and EP solutions. He is a co-founder and shareholder of YourRhythmics BV.

The remaining authors declare that the research was conducted in the absence of any commercial or financial relationships that could be construed as a potential conflict of interest.
